# Analysis of a cellular structure observed in the compound eyes of *Drosophila white; yata* mutants and *white* mutants

**DOI:** 10.1242/bio.047043

**Published:** 2020-01-13

**Authors:** Eri Arimoto, Yutaro Kawashima, Taein Choi, Mami Unagami, Shintaro Akiyama, Mizuki Tomizawa, Hiroyuki Yano, Emiko Suzuki, Masaki Sone

**Affiliations:** 1 Department of Biomolecular Science, Faculty of Science, Toho University, Funabashi 274-8510, Japan; 2 Technical Section, National Institute of Genetics, Mishima 411-8540, Japan; 3 Department of Gene Function and Phenomics, National Institute of Genetics, Mishima 411-8540, Japan; 4 Department of Genetics, SOKENDAI, Mishima 411-8540, Japan

**Keywords:** *Drosophila*, Eye, Photoreceptor, Bleb, Ageing, Autophagy

## Abstract

We previously identified the *Drosophila yata* mutant, which showed phenotypes including progressive vacuolization of the white-coloured compound eye, progressive shrinkage of the brain and a shortened lifespan. The *yata* gene was shown to be involved in controlling intracellular trafficking of the Amyloid precursor protein-like protein, which is an orthologue of Amyloid precursor protein, which is a causative molecule of Alzheimer's disease. In this study, we examined the phenotype of the compound eye of the *yata* mutant using electron microscopy and confocal microscopy. We found that abnormal cellular structures that seemed to originate from bleb-like structures and contained vesicles and organelles, such as multivesicular bodies and autophagosomes, were observed in aged *white; yata* mutants and aged *white* mutants. These structures were not observed in newly eclosed flies and the presence of the structures was suppressed in flies grown under constant dark conditions after eclosion. The structures were not observed in newly eclosed red-eyed *yata* mutants or wild-type flies, but were observed in very aged red-eyed wild-type flies. Thus, our data suggest that the observed structures are formed as a result of changes associated with exposure to light after eclosion in *white* mutants, *white; yata* mutants and aged flies.

## Abstract (precis)

**Summary:** We analysed a cellular structure formed by changes associated with exposure to light in the compound eyes of *Drosophila white* mutants, *white; yata* mutants and aged flies.

## INTRODUCTION

The compound eye of *Drosophila melanogaster* is a useful model system to study the mechanisms of development and maintenance of the visual system, because a variety of genetic techniques can be applied (Gaspar et al., 2018; Kumar, 2018; Lorincz et al., 2016; Montell, 2012; Perry et al., 2017). The compound eye is composed of approximately 800 unit eyes, or ommatidia, with each ommatidium containing eight photoreceptor neurons designated R1 to R8. Each photoreceptor neuron has densely stacked membrane structures consisting of microvilli called rhabdomeres, the membrane of which contains signalling proteins belonging to the phototransduction cascade, including rhodopsins. Because R7 is located at the distal side of the retina, while R8 is located at the proximal side of the retina just beneath R7, seven rhabdomeres can always be observed on tangential sections of an ommatidium. R7- and R8-level ommatidia can be distinguished by their distinct arrangements of rhabdomeres.

We previously identified *yata* mutants in *Drosophila* that showed progressive degenerative changes in both the compound eye and the central nervous system (Sone et al., 2009). Null mutants of *yata* exhibit progressive vacuolization of the compound eye in the *white* mutant background. This vacuolization phenotype was suppressed in flies grown under constant dark conditions after eclosion, suggesting the dependency of this phenotype on light. In addition to vacuolization, *yata* mutants showed developmental abnormalities in the compound eye, including the abnormal differentiation of photoreceptor neurons, which resulted in an increase or decrease in the number of rhabdomeres in each ommatidium. The compound eyes of *yata* mutants also showed defects in tissue polarity that resulted in a rotated spatial arrangement of ommatidia. *yata* mutants also showed progressive shrinkage of the brain and a notched wing phenotype and had a shortened lifespan. The function of *yata* in preventing degenerative changes in the nervous system is suggested to be evolutionarily conserved because mice with null mutation of the mammalian *yata* orthologue, *Scyl1*, showed motor neuron degeneration (Schmidt et al., 2007), and the human *Scyl1* gene has been identified as a causative gene in a genetic disease that causes liver failure, peripheral neuropathy, cerebellar atrophy and ataxia (Lenz et al., 2018; Schmidt et al., 2015; Shohet et al., 2019). In our previous study, we suggested that *yata* is involved in regulating anterograde intracellular trafficking of a subset of proteins. Our data suggest that *yata* regulates the localization of proteins, including APPL (Amyloid precursor protein-like) and Fasciclin II, whereas the localization of Synaptotagmin was not affected in *yata* mutants (Furotani et al., 2018; Sone et al., 2009). APPL is an orthologue of mammalian APP (Amyloid precursor protein), which is a causative molecule of Alzheimer's disease (Cassar and Kretzschmar, 2016; Poeck et al., 2012). The impaired localization of some proteins observed in *yata* mutants was suggested to be caused by impaired vesicular protein trafficking because aberrant accumulation of the COPII coat protein of secretory vesicles travelling from the endoplasmic reticulum to the Golgi was observed in *yata* mutants. The mammalian *yata* orthologue, SCYL1, was also suggested to play a role in the assembly of coated secretory vesicles including COPI-coated vesicles travelling from the Golgi to the endoplasmic reticulum (Burman et al., 2008; Hamlin et al., 2014). The phenotypes of *yata* mutants were exacerbated in flies carrying double-null mutations of *Appl* and *yata* and conversely rescued by the neuronal overexpression of *Appl*, suggesting that loss of function of *Appl* contributes to the phenotypes observed in *yata* mutants.

Our previous study showed that *yata* mutants exhibit progressive vacuolization in the compound eye in the *white* mutant background, whereas vacuolization was not generally observed in the red-eyed wild-type background (Sone et al., 2009). The compound eyes of wild-type *Drosophila* contain two types of pigments, drosopterins and ommochromes (Ewart and Howells, 1998; Lloyd et al., 1998). The compound eyes of wild-type *Drosophila* exhibit an intense red colour due to the presence of these pigments. The *white* gene encodes a protein that acts as an ATP-binding cassette (ABC) transporter involved in the uptake of precursors used for the synthesis of pigments into pigment granules (Ferreiro et al., 2017; Krstic et al., 2013). In null mutants of the *white* gene, pigments are absent from the compound eyes, and the eye colour is therefore white. Because these pigments limit the amount of light that enters the compound eye, the photoreceptor neurons in *white* mutants are exposed to much more light than those in wild-type flies (Hengstenberg and Götz, 1967). Another function of these pigments is to isolate rhabdomeres within each ommatidium to improve visual acuity. Indeed, *white* mutant flies show phenotypes caused by impairments in the phototransduction system. Moreover, a previous study showed that *white* mutants show progressive disorganization of the spatial arrangement of rhabdomeres, a progressive decrease in the sizes of rhabdomeres and progressive formation of vacuoles (Ferreiro et al., 2017).

In this study, we observed the phenotypes of the compound eyes of *yata* mutants using electron microscopy and confocal microscopy. We found that abnormal cellular structures containing organelles, such as multivesicular bodies and autophagosomes, formed in the compound eyes of aged *white; yata* mutants. We analyzed the identified cellular structures in different genotypes and ages.

## RESULTS

### Abnormal cellular structures were observed in the compound eyes of aged *white; yata* and aged *white* mutant flies

**Fig. 1.**
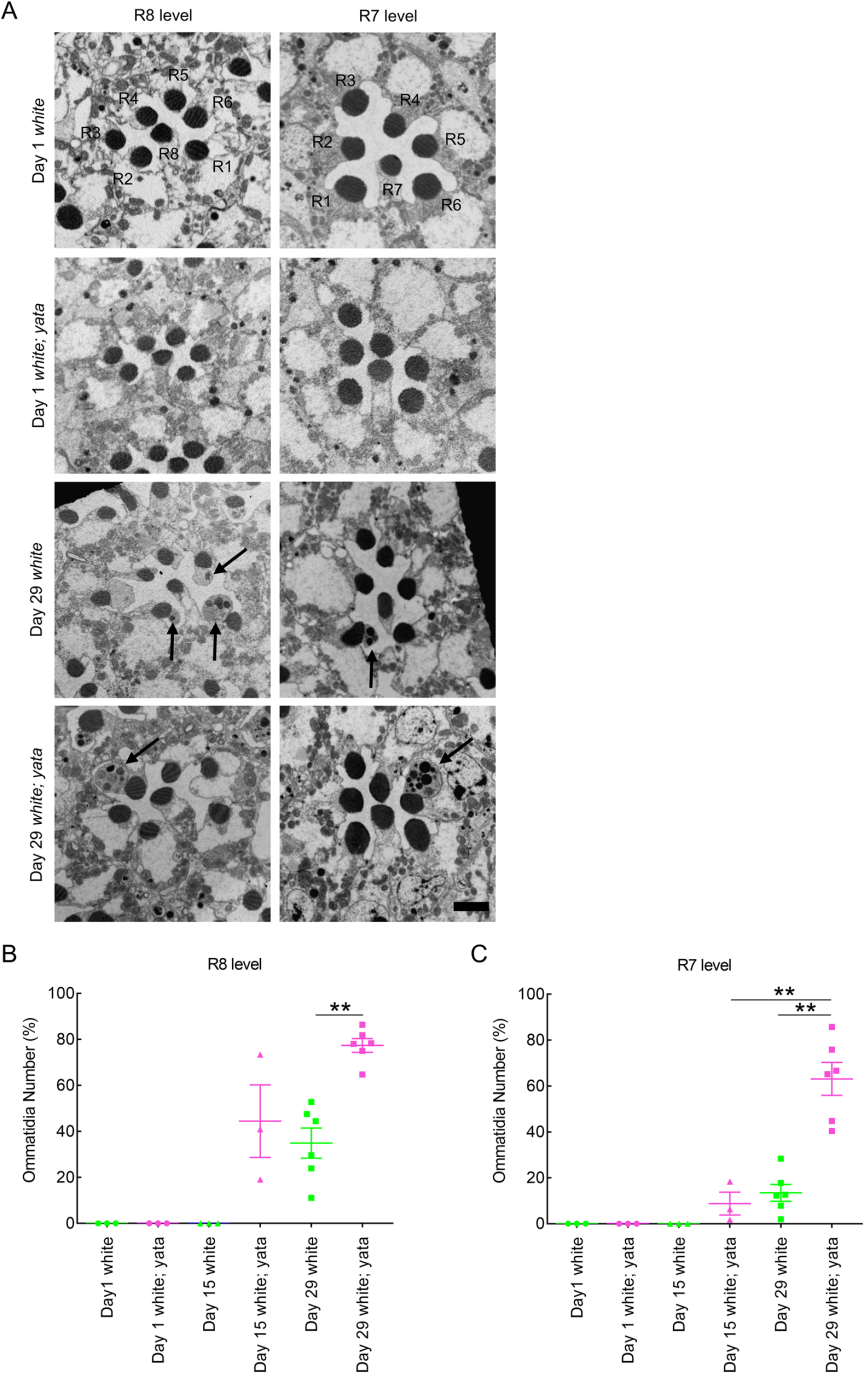
**Abnormal cellular structures were found in aged *white* and *white; yata* mutants.** (A) Electron micrographs of tangential sections of ommatidia at the R8 and R7 levels in flies with the indicated ages and genotypes are shown. In the *Drosophila* compound eye, each ommatidium contains eight photoreceptor neurons designated R1 to R8. Because R7 is located at the distal side of the retina while R8 is located at the proximal side just beneath R7, seven rhabdomeres can always be observed in tangential sections of an ommatidium. In day 29 *white* and *white; yata* mutants, abnormal cellular structures were found in the vicinity of rhabdomeres (arrows) at both the R8 and R7 levels. Scale bar: 2 μm. (B,C) The proportions of ommatidia at the R8 (B) and R7 (C) levels that contained at least one identified structure are plotted. Each dot represents data from one fly. The raw data are shown in Table S1. **P<0.01; Games-Howell test (B) and Tukey's test (C).

To clarify the mechanisms underlying the degenerative changes that occur in the nervous system of *yata* mutants, we observed the phenotypes of the compound eyes of *yata* mutants in detail using electron microscopy. Because a previous study showed that the compound eyes of *yata* mutants showed progressive vacuolization in the *white* mutant background, we first observed the compound eyes of aged day 29 *white; yata* mutants and compared them with those in day 29 *white* mutants, day 1 *white; yata* mutants and day 1 *white* mutants. The flies were reared under 12-h light/12-h dark conditions at 25°C. We prepared tangential sections of compound eyes from flies with these genotypes and observed both proximal ommatidia at the R8 level and distal ommatidia at the R7 level. We found that abnormal cellular structures were often observed in locations adjacent to rhabdomeres in ommatidia at both the R8 and R7 levels in day 29 *white; yata* mutants (Fig. 1A, arrows). The observed structures were typically 1 to 3 μm in diameter and often larger than rhabdomeres. We determined the sizes of 52 of the observed structures, which had an average diameter of 1.6 μm. The standard error of the mean was 0.17 μm. The observed structures were usually located near or opposite the central cavity. Similar structures were also observed in the day 29 *white* mutants. They were not observed in day 1 *white; yata* mutants or day 1 *white* mutants.

**Fig. 2.**
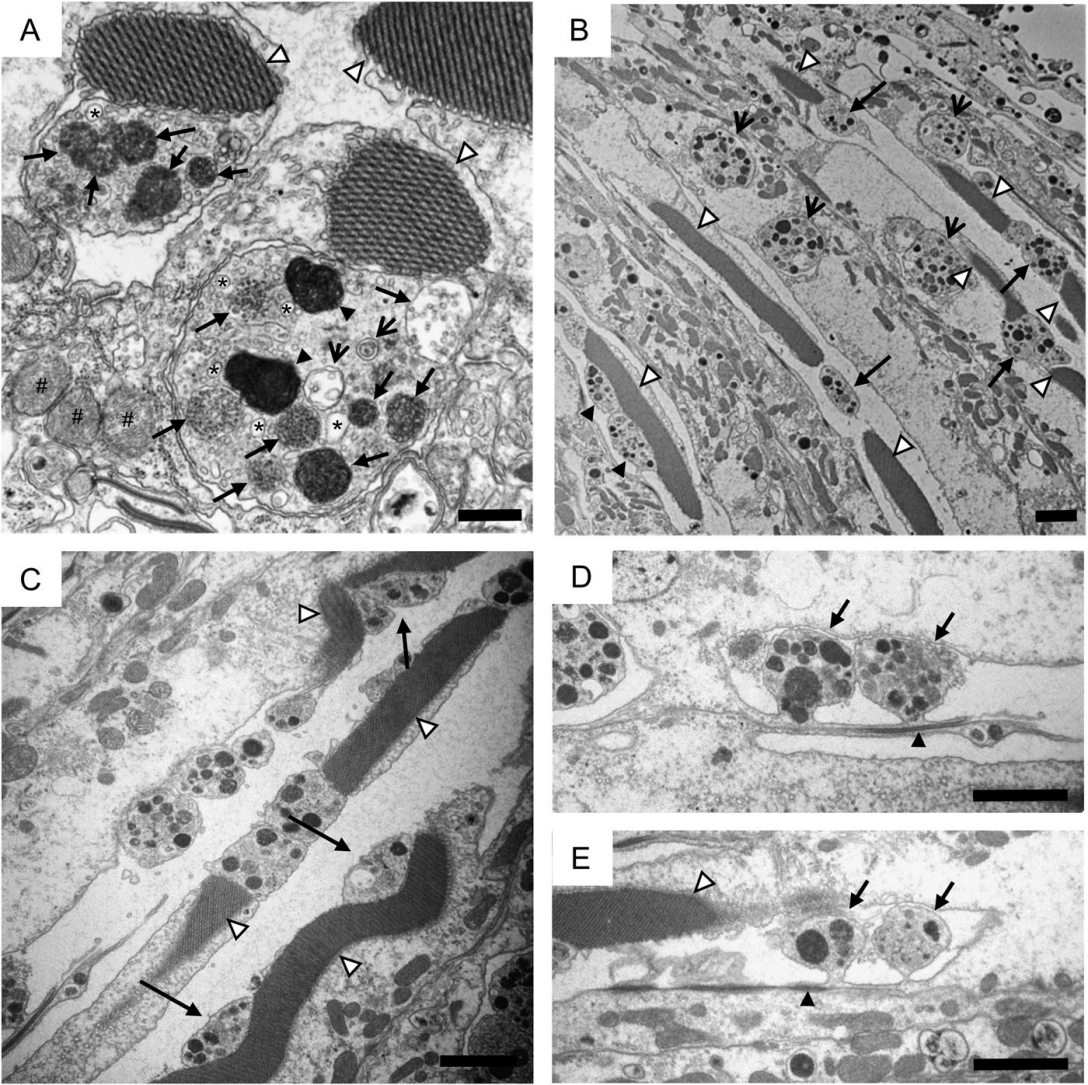
**The identified structures observed in day 29 *white; yata* mutants.** (A) Highly magnified image of the identified structures. An electron micrograph of a tangential section of an ommatidium at the R8 level is shown. Rhabdomeres are indicated by open arrowheads. The identified structures contained many vesicles, multivesicular bodies (arrows), electron-dense structures (filled arrowheads), vacuoles of various sizes (asterisks) and structures with double membranes (open arrows). Mitochondria that were found outside the identified structures are labelled with a hash. (B) An electron micrograph on a horizontal section of ommatidia is shown. Rhabdomeres are indicated by open arrowheads. The identified structures were found between rhabdomeres (arrows), lateral to rhabdomeres (filled arrowheads) and embedded in the cytoplasm of photoreceptor neurons (open arrows). (C) An electron micrograph of a horizontal section of ommatidia is shown. Rhabdomeres are indicated by open arrowheads. The identified structures occasionally caused rhabdomeres to twist (arrows). (D,E) Electron micrographs of horizontal sections of ommatidia are shown. The identified structures were observed to be bleb-like protrusions (arrows) near adherens junctions (filled arrowheads). A rhabdomere is indicated by an open arrowhead. Scale bars: 500 nm (A), 2 μm (B–E).

Close examination revealed that the structures were surrounded by a plasma membrane and contained vesicles, vacuoles of a variety of sizes (Fig. 2A, asterisks), and organelles such as multivesicular bodies (arrows), structures with double membranes (open arrows) and electron-dense structures (filled arrowheads). In contrast, mitochondria were not observed in the identified structures, although we carefully examined more than 50 of the structures. The identified structures were usually located adjacent to the rhabdomeres. Each rhabdomere was connected to the cytoplasm of the photoreceptor neuron from which it was derived, and different sides of rhabdomeres were adjacent to the identified structures. Rhabdomeres and the identified structures were often located side-by-side, but they also seemed to generally be separated by a plasma membrane. Even when the rhabdomere and the identified structure were derived from the same cell, the identified structure and the cytoplasm of the derivative photoreceptor neurons were separated by two plasma membranes and a space between the plasma membranes.

Next, we observed horizontal sections of ommatidia. Rhabdomeres in these horizontal sections exhibited a thin and long rod-like shape and spanned the proximal to the distal side of the retina. In day 29 *white; yata* mutants, the identified structures were often in between rhabdomeres (Fig. 2B, arrows). The identified structures were both connected to and separated from rhabdomeres or located lateral to rhabdomeres (Fig. 2B, arrows, arrowheads). Moreover, the identified structures sometimes seemed to cause rhabdomeres to which they were lateral to twist (Fig. 2C, arrows). In addition to their presence near rhabdomeres, the identified structures were also embedded in the cytoplasm of photoreceptor neurons (Fig. 2B, open arrows). We then carefully examined whether the identified structures were spatially connected to the cytoplasm of photoreceptor neurons. We found that the identified structures occasionally formed structures resembling blebs, which are spherical membrane protrusions associated with cell migration and apoptosis (Fig. 2D,E, arrows) (Charras and Paluch, 2008; Paluch and Raz, 2013). The bleb-like structures extended from the cytoplasm of cells facing the central cavity. Because only photoreceptor neurons surround the central cavity and the cells from which the bleb-like structures were derived contained rhabdomeres (Fig. 2E, open arrowhead), these cells were thought to be photoreceptor neurons. The bleb-like structures extended from locations near adherens junctions separating basolateral membranes and stalk membranes at the base of rhabdomeres (Fig. 2D,E, arrowheads) (Karagiosis and Ready, 2004; Satoh et al., 2013). These observations suggest that the identified structures originated from the bleb-like structures derived from the cytoplasm of photoreceptor neurons.

Next, we quantified the occurrence of the identified structures. Because the identified structures were more frequently observed in ommatidia at the R8 level than in ommatidia at the R7 level, we collected data for the R8 and R7 levels separately. We plotted the ratio of identified structure-containing ommatidia in each fly (Fig. 1B,C). Although the identified structures were not observed in day 1 *white* mutants and were also not observed in day 15 *white* mutants (Fig. 1B,C), the identified structures were observed in the compound eyes of day 29 *white* mutants, although the frequency was relatively low compared with that observed in day 29 *white; yata* mutants (Fig. 1B,C). Significantly fewer identified structures were found in ommatidia at both the R8 and R7 levels from day 29 *white* mutants than from day 29 *white; yata* mutants (*P*=0.001, one-way ANOVA and *P*=0.003, Games-Howell test for R8-level ommatidia; *P*=0.000, one-way ANOVA and *P*=0.000, Tukey's test for R7-level ommatidia). We further examined day 15 *white; yata* mutants and observed the identified structures in ommatidia at both the R8 and R7 levels (Fig. 1B,C); however, there were significantly fewer identified structures in ommatidia at the R7 level from these mutants than in those from day 29 *white; yata* mutants (*P*=0.000, Tukey's test). These data suggest that the compound eyes of newly eclosed flies do not contain the identified structures, but rather that the identified structures form in aged *white* and *white; yata* mutants. Additionally, the *yata* mutation enhances the formation of the identified structures.

**Fig. 3.**
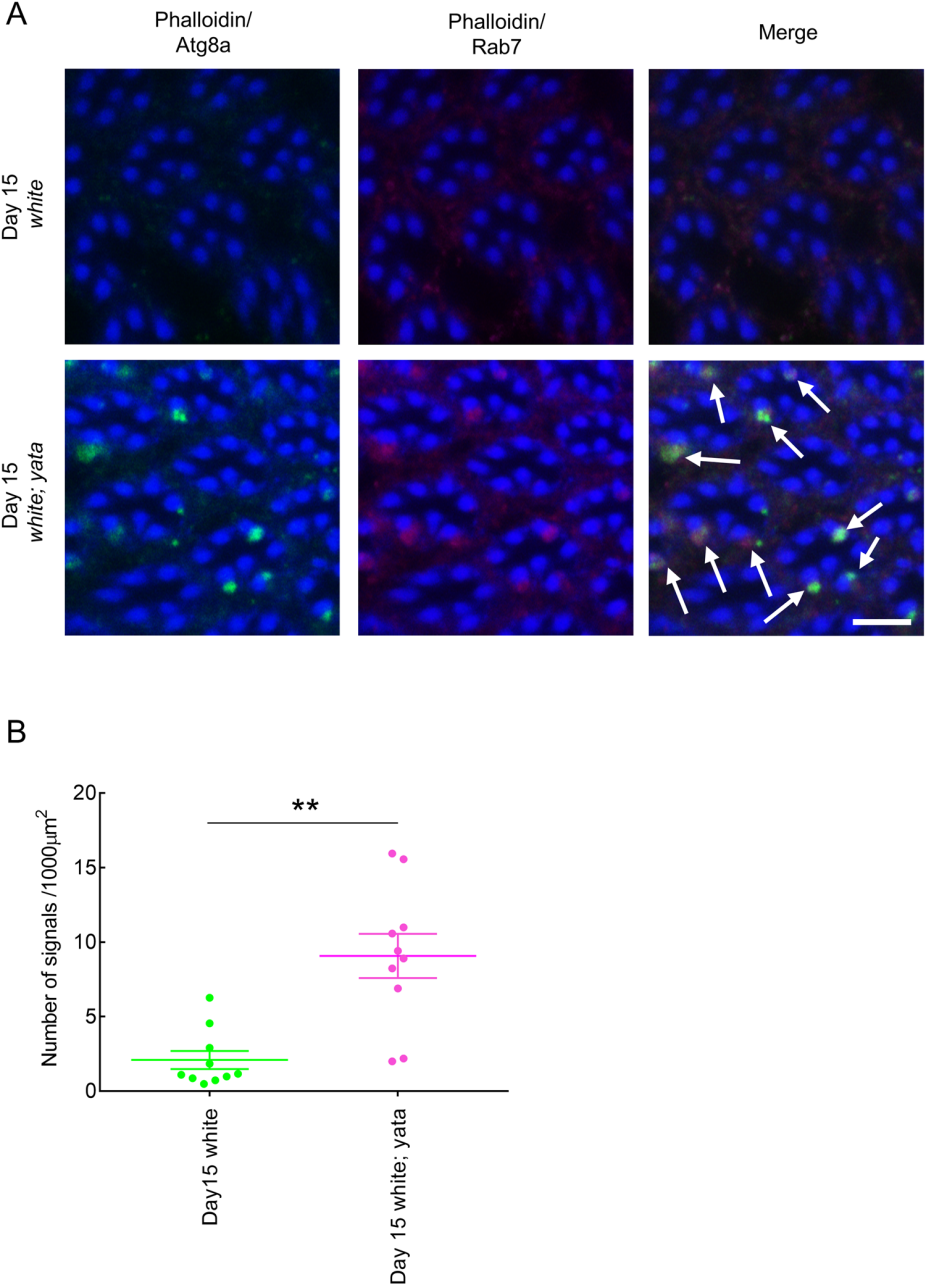
**Accumulation of autophagosomes and late endosomes in the day 15 *white; yata* mutants.** (A) R8-level retina of day 15 *white; yata* mutants stained with anti-Atg8a antibody (green) that labelled autophagosomes and anti-Rab7 antibody (magenta) that labelled late endosomes were observed in an image of a single plane by confocal microscopy. Accumulated autophagosomes and late endosomes (arrows) were observed near rhabdomeres labelled with phalloidin (blue). Relatively little accumulation was observed in day 15 *white* mutants. Scale bar: 5 μm. (B) Numbers of signals showing the accumulation of autophagosomes and late endosomes in the R8-level retinas of day 15 *white* and *white; yata* mutants are plotted. Each dot represents data from one fly. The raw data are shown in Table S1. **P<0.01; Mann–Whitney *U*-test.

Because the identified structures contained organelles, such as multivesicular bodies that seemed to be late endosomes, and structures with double membranes that may be autophagosomes, we examined the accumulation of markers of late endosomes and autophagosomes in day 15 *white; yata* and *white* mutants. We examined tangential optical sections of ommatidia at the R8 level by confocal microscopy. We found co-localized accumulation of the signals from anti-Atg8a antibody and anti-Rab7 antibody near the rhabdomeres labelled with phalloidin in the *white; yata* mutants (Fig. 3A). Anti-Atg8a antibody was originally raised against the mammalian GABARAP, GABARAPL1 and GABARAPL2 proteins and has previously been shown to specifically detect the *Drosophila* Atg8a protein in the immunostaining of the fat body of third instar larvae, as its signals disappeared in the *Atg8a* null mutants (Kim et al., 2015). Anti-Rab7 antibody has been shown to label late endosomes in the immunostaining of the *Drosophila* cells and tissues (Riedel et al., 2016). 3D reconstituted images also suggested that anti-Atg8a and anti-Rab7 signals often accumulated near rhabdomeres in the *white; yata* mutants (Fig. S1). Quantification of the number of signals suggested that the accumulation was more frequent in day 15 *white; yata* mutants than in day 15 *white* mutants (Fig. 3B; *P*=0.000, Mann–Whitney *U*-test). Our observations suggest that these signals represent the identified structures that were observed in the electron micrographs and that the identified structures contained autophagosomes and late endosomes.

### Formation of the identified structures was suppressed by constant dark conditions

**Fig. 4.**
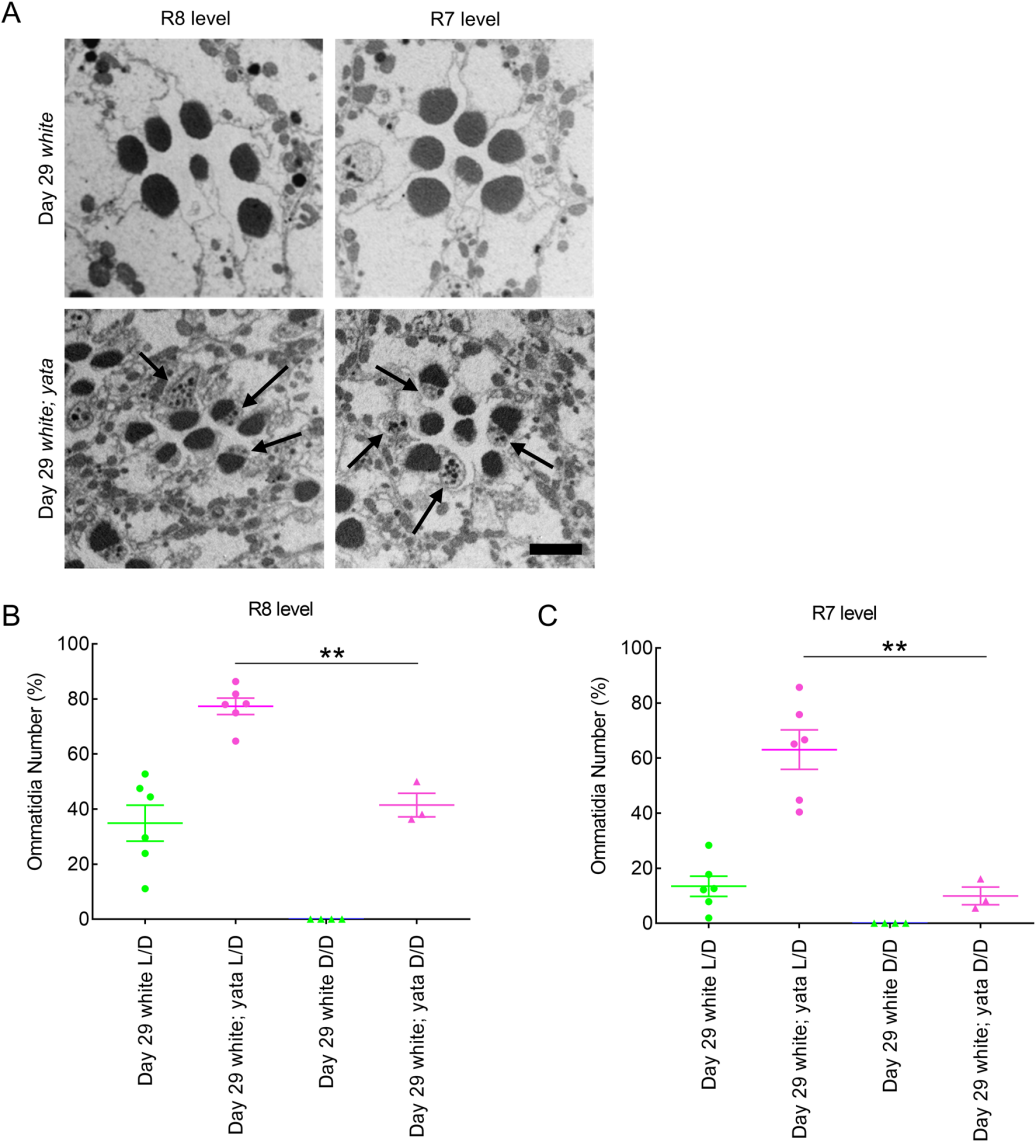
**Formation of the identified structures in flies reared under constant dark conditions after eclosion.** (A) Electron micrographs showing tangential sections of ommatidia at the R8 and R7 levels from flies of the indicated age and genotypes reared under constant dark conditions. In day 29 *white* mutants, formation of the identified structures in ommatidia at both the R8 and R7 levels was completely suppressed. In day 29 *white; yata* mutants, some ommatidia at both the R8 and R7 levels contained the identified structures (arrows). Scale bar: 2 μm. (B,C) The proportions of ommatidia at the R8 (B) and R7 (C) levels that contained at least one identified structure are plotted. Data collected from flies reared under 12-h light/12-h dark conditions (L/D) and those reared under constant dark conditions after eclosion (D/D) are compared. Each dot represents data from one fly. The raw data are shown in Table S1. **P<0.01; Games-Howell test (B) and Tukey's test (C).

Our previous data showed that progressive vacuolization is suppressed in *white; yata* mutants reared under constant dark conditions after eclosion (Sone et al., 2009). That the identified structures were not present in newly eclosed flies but rather formed in aged flies reared under 12-h light/12-h dark conditions (Fig. 1B,C) suggests that formation of the identified structures was caused by degenerative changes that proceeded after eclosion; thus, we examined whether exposure to light after eclosion affects formation of the identified structures. We reared *white* and *white; yata* mutants under constant dark conditions after eclosion and examined day 29 flies of each genotype. Formation of the identified structures in ommatidia at both the R8 and R7 levels was completely suppressed in *white* mutants reared under constant dark conditions after eclosion (Fig. 4A–C). However, in *white; yata* mutants, formation of the identified structures was not completely suppressed. Significantly fewer identified structures were observed in ommatidia at both the R8 and R7 levels in *white; yata* mutants reared under constant dark conditions than in flies reared under light/dark conditions (*P*=0.001, one-way ANOVA and *P*=0.008, Games-Howell test for R8-level ommatidia; *P*=0.000, one-way ANOVA and *P*=0.000, Tukey's test for R7-level ommatidia). These data suggest that exposure to light after eclosion is a causative factor in the formation of the identified structures that is completely and partly required for their formation in *white* and *white; yata* mutants, respectively.

### The identified structures were also observed in very aged red-eyed wild-type flies

**Fig. 5.**
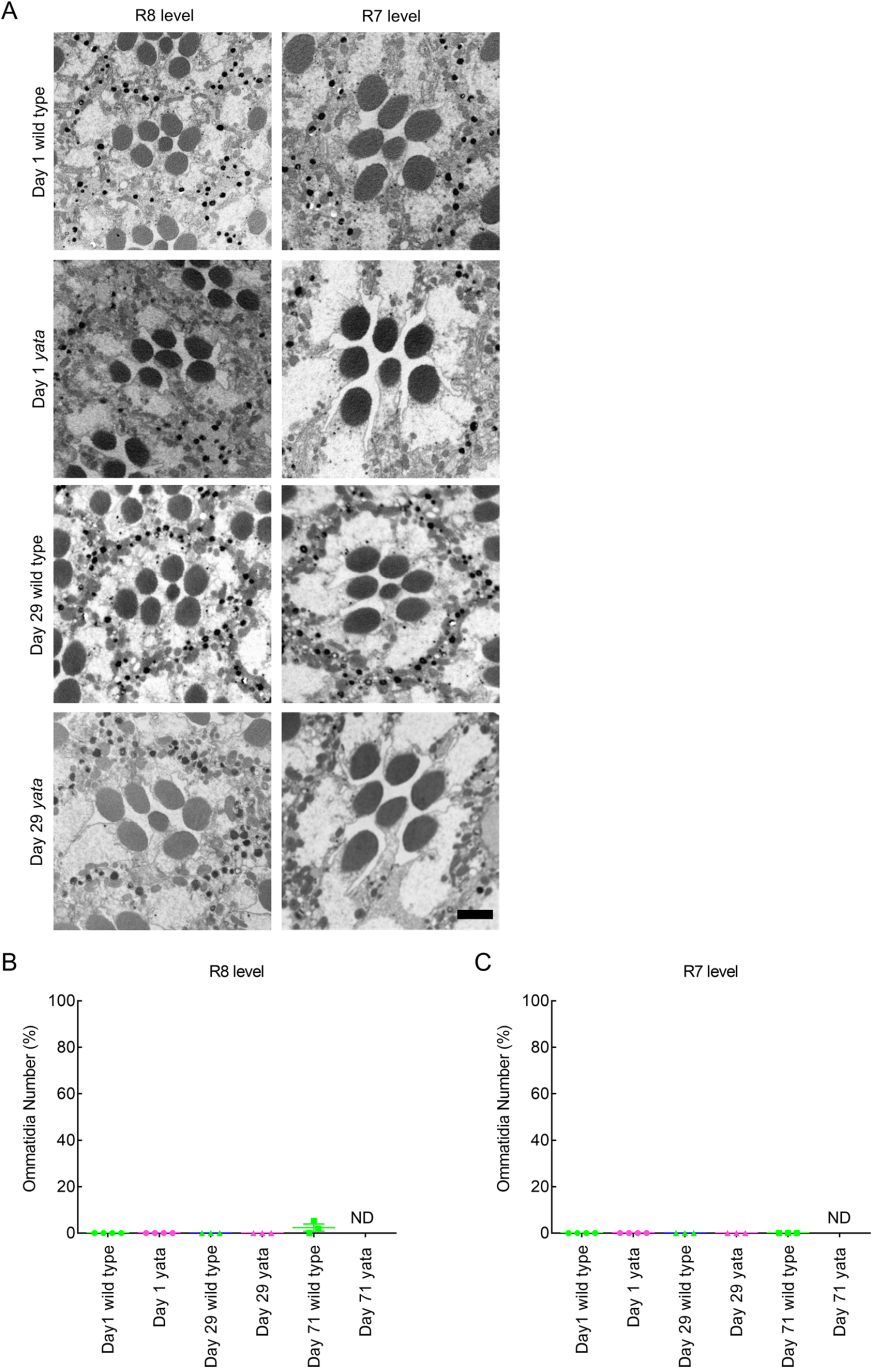
**The identified structures were not observed in red-eyed wild-type and *yata* mutant flies on days 1 and 29.** (A) Electron micrographs showing tangential sections of ommatidia at the R8 and R7 levels from flies of the indicated ages and genotypes. Scale bar: 2 μm. (B,C) The proportions of ommatidia at the R8 (B) and R7 (C) levels that contained at least one identified structure are plotted. Each dot represents data from one fly. The raw data are shown in Table S1. ND, not determined.

Our data suggest that the identified structures form after eclosion and that exposure to light after eclosion is a causative factor in their formation, especially in *white* mutants. Because the loss of function of pigments that protect photoreceptor neurons from excessive exposure to light may contribute to formation of the identified structures, we also examined whether the identified structures formed in red-eyed wild-type and *yata* mutant flies. We examined wild-type and *yata* mutant flies on days 1 and 29. The identified structures were not observed in ommatidia at either the R8 or R7 level in either wild-type or *yata* mutant flies (Fig. 5A–C). These data suggest that pigments protect the compound eyes of red-eyed wild-type and *yata* mutant flies.

**Fig. 6.**
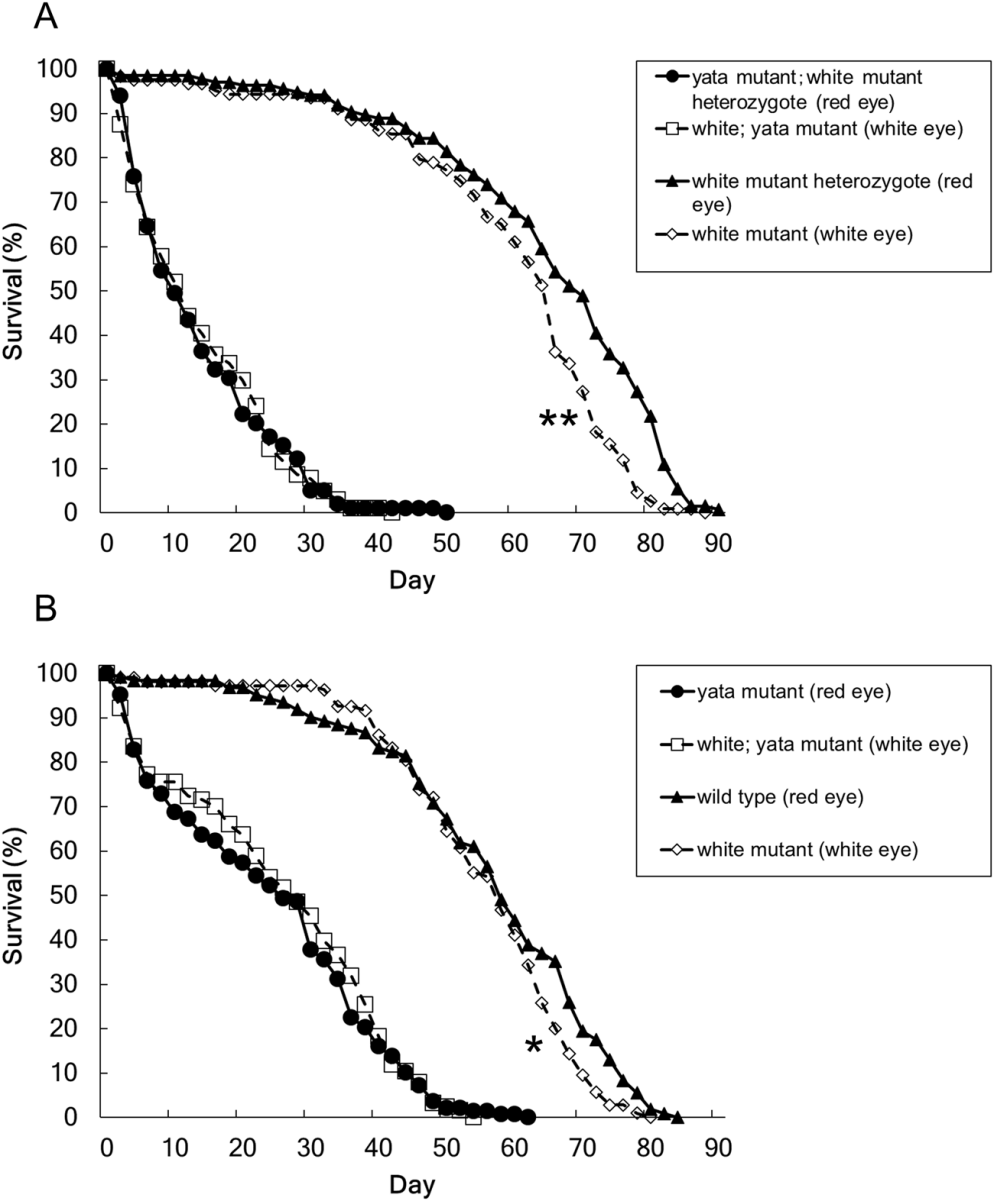
**Lifespan shortening in *yata* mutants was not affected by the *white* mutation.** The lifespans of (A) female and (B) male red-eyed and white-eyed *yata* mutants are shown. The *white* mutation did not enhance or rescue lifespan shortening observed in *yata* mutants. The lifespans of female and male red-eyed *white* mutant heterozygotes and white-eyed *white* mutants are also shown. The *white* mutation caused a slight shortening of the lifespans of both females and males. The numbers of flies examined were as follows: 99 (female, red eye, *yata* mutant), 104 (female, white eye, *yata* mutant), 137 (female, red eye, control), 125 (female, white eye, control), 145 (male, red eye, *yata* mutant), 127 (male, white eye, *yata* mutant), 128 (male, red eye, control) and 110 (male, white eye, control). *P<0.05, **P<0.01; log-rank test.

**Fig. 7.**
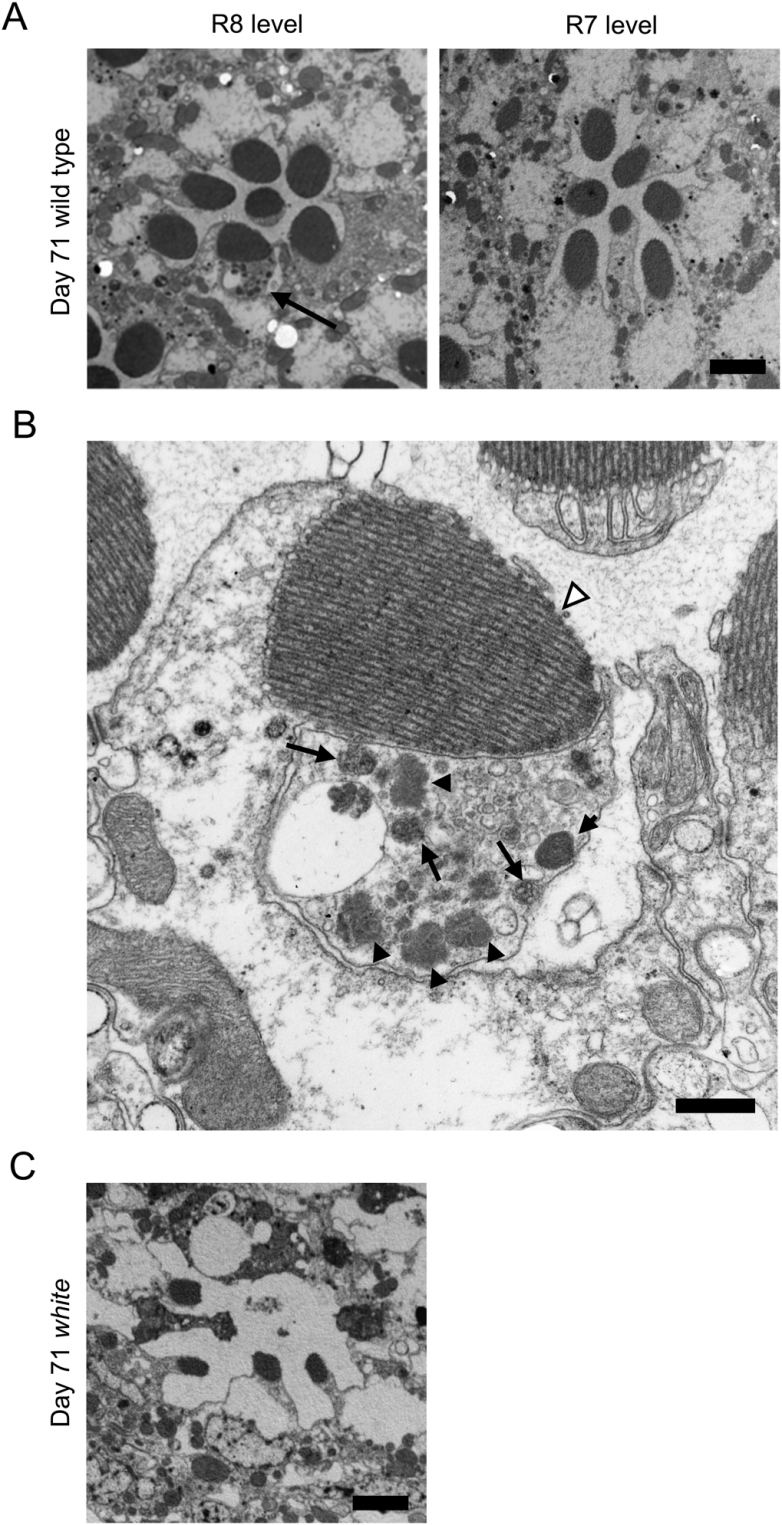
**The identified structures were observed in very aged day 71 wild-type flies.** (A) Electron micrographs showing tangential sections of ommatidia at the R8 and R7 levels from day 71 wild-type flies are shown. The identified structures were observed at low frequency in ommatidia at the R8 level (arrow). The identified structures were not observed in ommatidia at the R7 level. (B) Highly magnified image of the identified structure. An electron micrograph of a tangential section of an ommatidium at the R8 level is shown. The identified cellular structure contained many vesicles, multivesicular bodies (arrow) and electron-dense structures (filled arrowheads). A rhabdomere is indicated by an open arrowhead. (C) An electron micrograph of a tangential section of an ommatidium in a day 71 *white* mutant fly is shown. The tissue structure was severely damaged, although traces of rhabdomeres could be identified. Scale bars: 2 μm (A,C), 500 nm (B).

Next, we hypothesized that the cumulative effects of exposure to light induce formation of the identified structures in very aged flies, even in red-eyed flies, and we tested this possibility by examining day 71 wild-type flies reared under 12-h light/12-h dark conditions. Because the lifespan of wild-type flies is usually approximately 60–90 days (Fig. 6) (Nakayama et al., 2014), day 71 is near the limit of the wild-type fly lifespan. We found that cellular structures similar to the identified structures were observed at a low frequency in flies at this age (Figs 7A,B and 5B,C). We examined three wild-type flies at day 71 and found that two of them had the identified structures in ommatidia at the R8 level. The identified structures were not observed in ommatidia at the R7 level. These data suggest that the identified structures are also present in very aged wild-type flies. We also examined day 71 *white* mutant flies and found that the tissue structure was severely damaged and that many rhabdomeres had already been lost; furthermore, the identified structures were difficult to distinguish, even though traces of rhabdomeres could be identified (Fig. 7C).

### The *white* mutation did not affect the lifespan of *yata* mutants

The identified structures formed in white-eyed *yata* mutants at a high frequency (Fig. 1A–C), but red-eyed *yata* mutants did not show this phenotype (Fig. 5A–C). However, *yata* mutants showed progressive shrinkage of the brain and had a shortened lifespan. Previous studies have suggested that the *white* gene is involved in processes in addition to eye colour formation, including the metabolism of neurotransmitters in the brain (Borycz et al., 2008; Ferreiro et al., 2017; Krstic et al., 2013). Therefore, we tested whether the *white* mutation affects the lifespan phenotypes of control and *yata* mutant flies. To avoid the effects of differences in genetic background, we compared the lifespans of red-eyed and white-eyed control fly siblings and *yata* mutant siblings. The *white* gene is linked to the X chromosome, and the *yata* gene is linked to the third chromosome, which is an autosome. Female *white* mutant heterozygotes were crossed with male hemizygotes of the *white* mutants. Similarly, female *yata* mutant heterozygotes of *white* mutation heterozygotes were crossed with male heterozygotes of the *yata* mutants of the *white* mutant background. Among the progeny, we compared the lifespans of female red-eyed *white* heterozygotes and white-eyed *white* homozygotes, or female *yata* homozygous mutants of the red-eyed *white* heterozygotes and white-eyed *white* homozygotes. We also compared the lifespans of male red-eyed wild-type flies and white-eyed *white* hemizygotes, or male *yata* homozygous mutants with a red-eyed wild-type background to those of white-eyed *white* hemizygotes. The lifespans of red-eyed and white-eyed *yata* mutants were similar among both females and males (Fig. 6A,B). The differences were not statistically significant (log-rank test). These data suggest that the *white* mutation does not affect the lifespan-shortening phenotype observed in *yata* mutants. However, white-eyed control flies had a slightly shorter lifespan than red-eyed control flies (*P*=0.000 for females and *P*=0.041 for males, log-rank test). These data suggest that the *white* mutation slightly affected the lifespan of the control flies.

## DISCUSSION

We previously revealed that *Drosophila yata* mutants showed progressive degenerative changes in both their compound eyes and central nervous system (Sone et al., 2009). In this study, we observed the phenotypes of the compound eyes of *yata* mutants in fine detail using electron microscopy. Our observations led us to identify abnormal cellular structures located in the vicinity of rhabdomeres in aged day 15 and day 29 *white; yata* mutants. In tangential sections, rhabdomeres were connected to the cytoplasm of the cells from which they were derived, and a different side of these rhabdomeres was often also attached to the identified structures. Although the identified structures were surrounded by a plasma membrane, they were often attached to rhabdomeres. The identified structures were usually located near or opposite the central cavity. In horizontal sections, the identified structures were often found in between rod-like rhabdomeres, and were found to be both connected to and separated from rhabdomeres. They were also found lateral to rhabdomeres and embedded in the cytoplasm of photoreceptor neurons, and were also occasionally observed to cause twisting of the rhabdomeres. Because the identified structures seem to adhere to rhabdomeres, there may be adhesive mechanisms between the identified structures and rhabdomeres. In addition, in some electron micrographs, the identified structures were observed to be bleb-like structures that extended from the cytoplasm of photoreceptor neurons from locations near the adherens junction. These observations suggest that the identified structures originate as bleb-like structures from the cytoplasm of photoreceptor neurons and may migrate into the location of rhabdomeres or the cytoplasm of photoreceptor neurons. The identified structures were filled with vesicles, vacuoles and membranous organelles including multivesicular bodies that appeared to be late endosomes, structures with double membranes and electron-dense structures. Our analysis using confocal microscopy revealed the accumulation of anti-Atg8a and anti-Rab7 signals near the rhabdomeres in the day 15 *white; yata* mutants. Anti-Rab7 antibody has been shown to label late endosomes (Riedel et al., 2016). Anti-Atg8a antibody has previously been shown to specifically detect the *Drosophila* Atg8a protein in immunostaining of the fat body of third instar larvae. Although previous studies showed that anti-Atg8 antibody can label aggregates of ectopically overexpressed Atg8 protein or Atg8 protein incorporated into protein aggregates in cultured cells (Kuma et al., 2007; Pircs et al., 2012), the anti-Atg8a signal observed in the *white; yata* mutants seems to represent the accumulation of autophagosomes, as it is consistent with our observation that the identified structures contain structures with double membranes on the electron microscopy. Thus, these data suggest that the identified structures contained autophagosomes and late endosomes. Moreover, electron-dense structures observed in the identified structures appeared to be similar to lysosomes. These findings suggest that the identified structures may contain organelles involved in protein degradation pathways, such as the autophagy-lysosome system.

In addition to their presence in day 15 and day 29 *white; yata* mutants, the identified structures were found in day 29 *white* mutants. However, the identified structures were not found in day 1 *white; yata* or *white* mutants. These structures may be similar to ‘multivesicular body-like bodies’ previously observed in aged day 21 *white* mutants (Stark et al., 1988). These ‘multivesicular body-like bodies’ were found to occasionally form a peninsula-like structure that extended from the cytoplasm of a photoreceptor neuron near the adherens junction, and this observation is consistent with the bleb-like form of the identified structures we observed. The identified structures were not found in red-eyed *yata* mutant and wild-type flies on days 1 and 29. Formation of the identified structures was completely suppressed in day 29 *white* mutants reared under constant dark conditions after eclosion. These findings suggest that formation of the structure in *white* mutants is caused by changes in photoreceptor neurons associated with excessive exposure to light after eclosion because pigment granules were absent in the *white* mutants. In addition, our data showed that the identified structures also formed in very aged day 71 red-eyed wild-type flies. These changes may be a component of the changes associated with ageing in the compound eyes of flies.

Significantly more identified structures were observed in the day 29 *white; yata* mutants than in the day 29 *white* mutants reared under 12-h light/12-h dark conditions. The identified structures were observed in the day 15 *white; yata* mutants, but not in the day 15 *white* mutants. Therefore, the *yata* mutation may enhance formation of the identified structures, although whether the *yata* mutation enhances formation of the identified structures in flies with a red-eyed background is unclear. The photoreceptor neurons of *yata* mutants, at least those in the *white* mutant background, may be more vulnerable to changes associated with exposure to light. In addition, formation of the identified structures was not completely suppressed in day 29 *white; yata* mutants reared under constant dark conditions after eclosion, although no identified structures were observed in day 29 *white* mutants reared under constant dark conditions after eclosion. Therefore, the *yata* mutation may enhance the mechanisms that lead to formation of the identified structures, even in flies reared under constant dark conditions after eclosion. The loss of such *yata*-dependent mechanisms may cause formation of the identified structures in the background of a null mutation of *white* or in flies in which pigment granules have been lost. Diverse phenotypes in addition to a white eye colour have been described in *white* mutants, suggesting a wide range of molecular functions for the *white* gene (Ferreiro et al., 2017; Krstic et al., 2013). Although why *white* mutation enhanced formation of the identified structures in *white; yata* mutants reared under constant dark conditions after eclosion is unclear, this effect may be related to the fact that pigment granules are lysosome-like organelles (Lloyd et al., 1998) and that several genes involved in the biogenesis of pigment granules also play a role in lysosome trafficking (Lőrincz et al., 2016). Notably, giant multivesicular structures with diameters greater than 3 μm have been observed in the pigment cells of mutants of the *deep orange* gene, which encodes an endosomal protein required for normal eye colour (Sevrioukov et al., 1999).

Our data suggest that the identified structures are more frequently observed in ommatidia at the R8 level than in those at the R7 level regardless of genotype or age. These observations suggest that the identified structures form more frequently in a proximal position in the retina than in a distal position in the retina. Although the reason for this location-dependent differential vulnerability is unknown, it may be related to a previous observation that retinal degeneration processes occurred more rapidly in proximal positions remote from the nucleus than in distal positions in *rdgA* mutants (Matsumoto et al., 1988). Therefore, the difference in the frequency of the identified structures between the ommatidia at the R8 and R7 levels may be related to the susceptibility of photoreceptor neurons to degenerative changes.

Previous studies suggested that *yata* is involved in regulating the intracellular trafficking of a subset of proteins including APPL and Fasciclin II (Furotani et al., 2018; Sone et al., 2009). However, it has also been suggested that *yata* does not control the localization of all membrane-bound and secreted proteins transported via vesicular trafficking because the localization of Synaptotagmin was not affected by *yata* mutation. In addition, *yata* may also control the trafficking of some unidentified proteins. Therefore, the loss of *yata* may enhance formation of the identified structures by impairing the localization of APPL, Fasciclin II or other unidentified target proteins or an unidentified molecular function of *yata*.

In conclusion, we identified a cellular structure that contains vesicles, autophagosomes and multivesicular bodies in the compound eyes of aged *white; yata* mutants, aged *white* mutants and very aged wild-type flies. Our data suggest that these structures are formed as a result of changes associated with exposure to light after eclosion in *white* mutants, *white; yata* mutants and aged flies. Further exploration of the mechanisms of formation of the identified structures may clarify their physiological significance.

## MATERIALS AND METHODS

### Fly genetics

Flies were raised on yeast-cornmeal medium (7.5% cornmeal, 3.8% yeast, 9.4% glucose, 3.0% wheat germ, 0.24% n-butyl p-hydroxybenzoate, 0.09% calcium chloride, 1.1% potassium tartrate hemihydrate and 0.9% agar). All fly stocks were maintained at 25°C under a 12:12 h light-dark cycle of normal laboratory illumination at approximately 1000 lux (LM-332 light metre, As-one), except in the experiment using constant dark conditions. For constant dark conditions, flies were reared in a can and food was exchanged under a dim red safety light. The *w^1118^* null mutant strain was used as a *white* mutant. We used *w^1118^* as a control strain and all of the flies used were isogenized to the same *w^1118^* control strain by consecutive backcrossing of more than three generations. We isogenized Canton-S flies to the *w^1118^* control strain and used them as the red-eyed wild-type flies. To measure lifespan, 1 to 20 flies were reared at 25°C in a standard plastic vial. We determined the number of live flies and assessed the condition of the medium every day. Flies were transferred to a new vial when the condition of the medium deteriorated or 7 days had passed since the flies had been transferred to the vial. Lifespan data were collected from one consecutive series of experiments.

### Electron microscopy

Thin sections were prepared for electron microscopy as previously described (Matsumoto et al., 1988; Suzuki and Hirosawa, 1994). The heads of female flies were collected in the daytime, cut longitudinally into halves and fixed in fixative (2.5% glutaraldehyde and 2% formaldehyde buffered with 0.1 M sodium cacodylate to pH 7.3) for more than 2 h at room temperature. They were then rinsed with 4% sucrose solution in the same buffer for 5 min three times on ice, post-fixed with 1% osmium tetroxide in the same buffer for 1.5 h on ice and rinsed with distilled water for 5 min three times on ice. Then the samples were stained *en bloc* with 0.5% uranyl acetate in distilled water for 1 h on ice, dehydrated in 50% ethanol, followed by 70% ethanol and then 90% ethanol each for 5 min on ice, and then in dry ethanol for 5 min three times, and in propylene oxide for 5 min twice. The samples were then mixed with propylene oxide and Epon at a ratio of 1:1 overnight and embedded in Epon. Semithin sections (500 nm) were cut with a diamond knife using an ultramicrotome (Leica Ultracut S). By staining the sections with Toluidine Blue and examining them, we obtained sample block surfaces that contained both R7- and R8-level ommatidia. For each sample, we chose a surface that contained more than 20 each of R7- and R8-level ommatidia. Then, we cut the thin sections (70 nm). The sections were stained with 2% uranyl acetate solution for 5 min and then Reynolds lead citrate solution for 5 min. Electron micrographs were acquired with a JEOL JEM-1010 electron microscope. Photos were taken of entire regions of single sections that covered the compound eye. All of the ommatidia in the section were examined and counted. We examined all ommatidia categorized at the R8 or R7 level or observed to contain the identified structure. If a photoreceptor neuron in an ommatidium had at least one identified structure, the ommatidium was considered to contain the identified structure. Structures surrounded by a plasma membrane that contained more than one multivesicular body or electron-dense structure were defined as the identified structures.

### Confocal microscopy

Dissection and immunostaining of adult retina were performed essentially as previously described (Midorikawa et al., 2010). The heads of female flies were collected in the daytime and cut longitudinally into halves. Then, the retinas were cut tangentially with scissors (Vannas scissors, World Precision Instruments) in phosphate-buffered saline (PBS). The retinas were fixed in 4% paraformaldehyde in PBS for 1 h on ice and washed three times for 10 min in PBS with 0.4% Triton X-100. The samples were incubated in rabbit anti-Atg8a antibody (Abcam, ab109364, diluted 1:200) (Kim et al., 2015) and mouse anti-Rab7 antibody (Developmental Studies Hybridoma Bank, Iowa University, diluted 1:50) (Riedel et al., 2016) in PBS with 0.4% Triton X-100 overnight at 4°C. They were washed three times for 10 min in PBS with 0.4% Triton X-100 and then incubated with Cy3-conjugated anti-mouse IgG antibody (Jackson, 715-165-150, diluted 1:100), Cy5-conjugated anti-rabbit IgG antibody (Jackson, 711-175-152, diluted 1:100) and fluorescein phalloidin (Fujifilm Wako Pure Chemicals, 068-06261, 66 nmol/l) in PBS with 0.4% Triton X-100 for 1 h at room temperature. They were washed three times for 10 min in PBS with 0.4% Triton X-100 and then mounted in Vectashield with DAPI (Vector, H-1200). Images were collected with a confocal microscope (Olympus, FV-1000) equipped with a UPlanSApo 60×/1.35 oil-immersion objective with 2× magnification and optical resolutions of 0.223 μm for the X- and Y-axes and 1.028 μm for the Z-axis. Three repetitively scanned images were integrated with the Kalman method. All signals in triple-stained samples were scanned separately. The same scanning conditions were used to acquire images of samples from flies of all genotypes to observe Atg8a and Rab7 signals. To obtain a 3D-reconstituted image, 21 optical sections were scanned with a 1.0 μm interval and images were reconstituted with FV10-ASW 3.1 software (Olympus). Images were processed with Adobe Photoshop Elements 11. The contrast and brightness were adjusted across entire images, and similar adjustments were applied to images from flies of all genotypes. To quantify signals indicating the accumulation of autophagosomes and late endosomes, double-stained signals in which the diameter was larger than 1.0 μm were counted by observers blinded to the genotype. The areas of the measured regions were quantified with Cellsens Dimension software (Olympus).

### Statistical analysis

All of the data are shown as the mean±s.e.m. We performed statistical analysis using SPSS 24 software. We used a parametric test unless the data were predicted to exhibit a non-normal distribution. Otherwise, we used a nonparametric test. When the averages from two samples in the confocal microscopy experiment were compared, we used the Mann–Whitney *U*-test because the data were predicted to be significantly non-normally distributed by the Kolmogorov–Smirnov test. When the averages from more than two samples in the electron microscopy experiments were compared, we used one-way ANOVAs with post hoc tests. To choose the post hoc test, we compared the variance in the data by Levene's test. When the variance was predicted to be different, we used the Games-Howell test. Otherwise, we used Tukey's test. To compare lifespan data, we used the log-rank test.

## Supplementary Material

Supplementary information
