# Supplementary material for: Analysis of a cellular structure observed in the compound eyes of Drosophila white; yata mutants and white mutants
Source: Biol Open. 2020 Jan 13;9(1):bio047043. doi: 10.1242/bio.047043 (PMC6994944; doi:10.1242/bio.047043)
Supplement: Supplementary information [file biolopen-9-047043-s1.pdf]

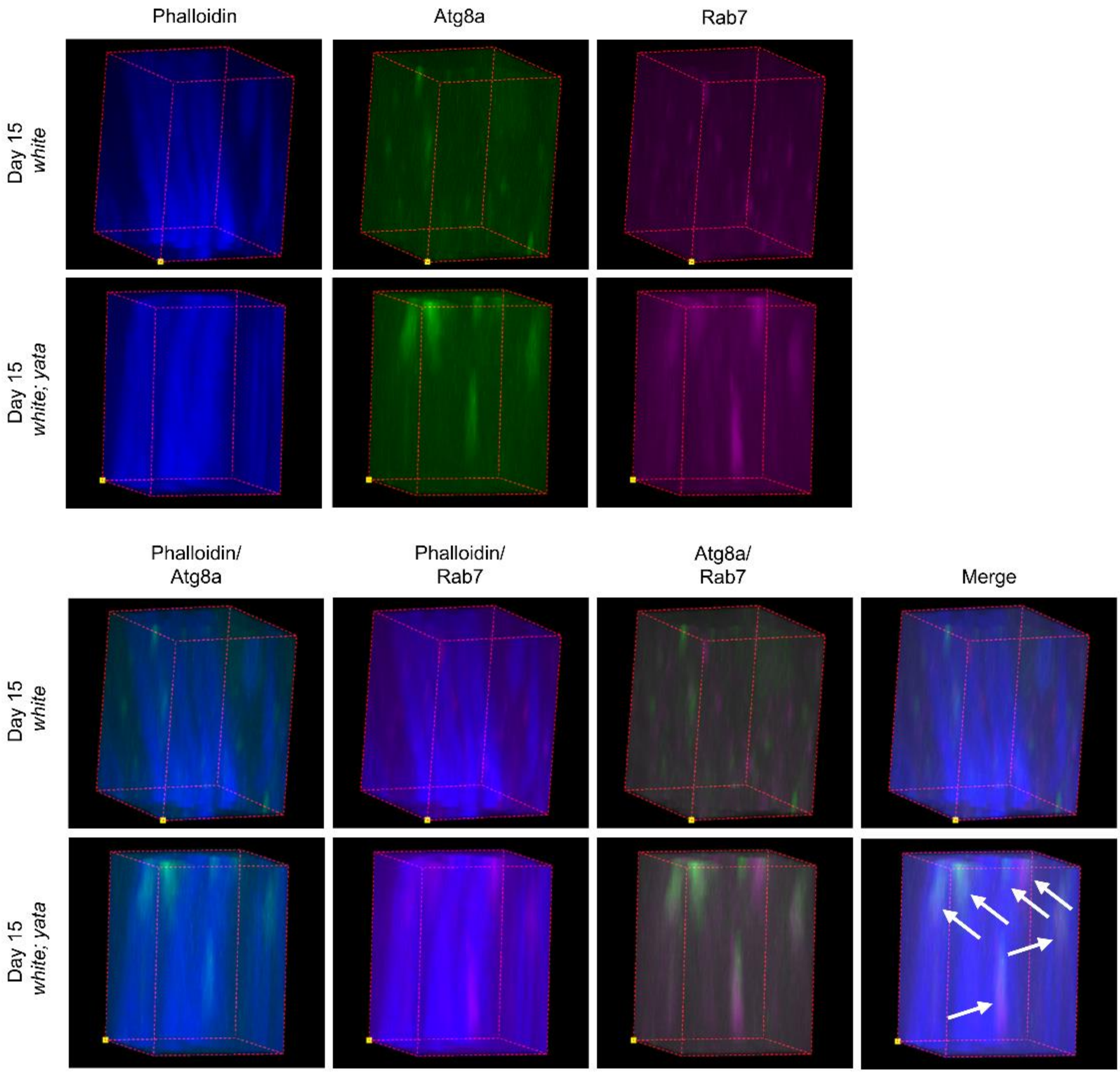

Fig. S1. 3D reconstituted image of the R8-level retinas of the day 15 *white; yata* and *white* mutants stained with anti-Atg8a and anti-Rab7 antibodies. An Image of an ommatidium is shown. Signals from the anti-Atg8a antibody are shown in green, and signals from the anti-Rab7 antibody are shown in magenta. Rhabdomeres were labelled with phalloidin (blue). Twenty-one optical sections with a 1.0 µm step size were scanned and reconstituted. The resctangles are 15.0 µm (*white*) or 14.5 µm (*white; yata*) in size along the X-axis and Y-axis and 21.0 µm in size along the Z-axis. Strong overlapping or adjacent signals for Atg8a and Rab7 (arrows) were observed near rhabdomeres in the *white; yata* mutant.

Biology Open • Supplementary information

Table S1

Click here to Download Table S1

Biology Open • Supplementary information
